# Leukemia Inhibitory Factor (LIF) Overexpression Increases the Angiogenic Potential of Bone Marrow Mesenchymal Stem/Stromal Cells

**DOI:** 10.3389/fcell.2020.00778

**Published:** 2020-08-14

**Authors:** Girlaine Café Santos, Daniela Nascimento Silva, Vitor Fortuna, Brysa Mariana Silveira, Iasmim Diniz Orge, Thaís Alves de Santana, Gabriela Louise Sampaio, Bruno Diaz Paredes, Ricardo Ribeiro-dos-Santos, Milena Botelho Pereira Soares

**Affiliations:** ^1^Gonçalo Moniz Institute, Oswaldo Cruz Foundation, Salvador, Brazil; ^2^Health Institute of Technology, SENAI-CIMATEC, Salvador, Brazil; ^3^Health Sciences Institute, Federal University of Bahia, Salvador, Brazil; ^4^Research D’Or Institute, Rio de Janeiro, Brazil; ^5^National Institute of Science and Technology for Regenerative Medicine, Rio de Janeiro, Brazil

**Keywords:** mesenchymal stem/stromal cells, genetic modification, LIF, proangiogenico factors, angiogenesis

## Abstract

Mesenchymal stem/stromal cells (MSCs) have the ability to secrete bioactive molecules, exerting multiple biological effects, such as tissue regeneration, reduction of inflammation, and neovascularization. The therapeutic potential of MSCs can be increased by genetic modification to overexpress cytokines and growth factors. Here we produced mouse MSCs overexpressing human leukemia inhibitory factor (LIF) to assess their proangiogenic potential *in vitro* and *in vivo*. Mouse bone marrow-derived MSCs were transduced by using a second-generation lentiviral system to express human LIF. Leukemia inhibitory factor expression was confirmed by RT-qPCR and by ELISA, allowing the quantification of the transcript and secreted protein, respectively. Flow cytometry analysis and trilineage differentiation assay showed that the MSC_LIF cell line maintained the immunophenotype and a multipotency characteristic of MSCs. The immunosuppressive activity of MSC_LIF was confirmed using a lymphoproliferation assay. Moreover, gene expression analysis demonstrated upregulation of genes coding for strategic factors in the neovascularization process, such as angiogenin, IL-8, MCP-1, and VEGF, and for the perivascular cell markers αSMA, Col4a1, SM22, and NG2. To evaluate the pro-angiogenic potential of MSC_LIF, we first tested its effects on endothelial cells obtained from umbilical vein in a scratch wound healing assay. Conditioned medium (CM) from MSC_LIF promoted a significant increase in cell migration compared to CM from control MSC. Additionally, *in vitro* tube formation of endothelial cells was increased by the presence of MSC_LIF, as shown in microvessel sprouting in aortic ring cultures. Finally, an *in vivo* Matrigel plug assay was performed, showing that MSC_LIF were more potent in promoting *in vivo* angiogenesis and tissue vascularization than control MSCs. In conclusion, LIF overexpression is a promising strategy to increase the proangiogenic potential of MSCs and sets precedents for future investigations of their potential applications for the treatment of ischemic diseases and tissue repair.

## Introduction

The potential of mesenchymal stem/stromal cells (MSCs), a cell population easily obtainable from different sources in the adult organism, has been intensely explored in the past decades for the development of cell and gene therapies. The main therapeutic properties of MSCs are attributed to their ability to secrete an array of soluble bioactive molecules, such as cytokines and growth factors, important in the regulation of several biological processes, including inflammation and fibrosis, cell growth, tissue repair and angiogenesis (Caplan and Dennis, 2006; Parekkadan et al., 2007; Kim et al., 2019). Several studies have investigated the therapeutic potential of MSCs for ischemic diseases, stimulating not only the development of MSCs-based therapies but also the identification of factors and molecules responsible for their pro-angiogenic potential (Gearing et al., 1987). However, differences in potency among cell populations and heterogeneity in the MSC expression profile represent a limitation of their therapeutic use (Murphy et al., 2013).

Leukemia inhibitory factor (LIF), a highly pleiotropic cytokine belonging to the interleukin-6 superfamily, was initially described as an inhibitory factor for myeloid leukemic cells proliferation (Auernhammer and Melmed, 2000). Currently, it is known that LIF acts regulating cell proliferation, differentiation and survival, as well as maintaining the state of pluripotency and self-renewal of stem cell populations, including embryonic stem cells and MSCs (Williams et al., 1988; Jiang et al., 2002; Metcalf, 2003). Other biological properties of LIF include induction of bone remodeling, neuroprotection, cardiac regeneration and regulation of hematopoiesis (Pepper et al., 1995). The role of LIF in angiogenesis has not been completely elucidated, and different studies have shown contradictory responses. The direct effect of LIF on endothelial cells appears to inhibit their angiogenic capacity, while populations of stem cells are stimulated to secrete important proangiogenic growth factors in the presence of LIF, contributing to neovascularization. Thus, the levels of LIF secretion by MSCs may be relevant for the therapeutic effects of these cells (Chen et al., 2014; Nicola and Babon, 2015).

Genetic manipulation of MSCs has been tested as a strategy to generate cells expressing factors capable of increasing or potentiating the biological activities of MSCs, such as migration, survival after transplantation, and secretion of molecules of interest for the treatment of different disease settings (D’souza et al., 2015). This can be achieved by genetic engineering using different viral vectors for transduction or by plasmid transfection. Here we used a second-generation lentiviral system to produce mouse bone marrow-derived MSCs overexpressing LIF (MSC_LIF) to assess their potential on angiogenesis.

## Materials and Methods

### Isolation and Culture of Mouse Bone Marrow MSC

Bone marrow-derived MSCs were obtained from GFP transgenic C57Bl/6 male mice aged 4-8 weeks. The animals were kept in the animal facility of the Center for Biotechnology and Cell Therapy of Hospital São Rafael (Salvador, Brazil), with access to food and water *ad libitum*. The procedures for obtaining the cells were approved by the ethics committee for animal use of Hospital São Rafael (CEUA-HSR 007/18). Bone marrow cells obtained from the tibiae and femurs by flushing were centrifuged at 300 × *g* for 10 min. The pellet was resuspended in 10 ml of Dulbecco’s Modified Eagle’s Medium (DMEM), supplemented with 10% fetal bovine serum (FBS) and 1% penicillin/streptomycin (all Thermo Fisher Scientific, Waltham, MA, United States) and cultured in plastic flasks in incubator at 37°C with 5% atmospheric CO_2_. After two days, the culture medium was completely changed and the non-adherent cells were removed. The adhered cells were cultured at 37°C with 5% atmospheric CO_2_ and the medium was changed every 3 days until the monolayer reached 90% confluence. The MSCs were detached using a trypsin-EDTA 0.25% solution (Thermo Fisher Scientific) for expansion and use for transduction.

### Lentiviral Production and Transduction of MSCs

**FIGURE 1:**
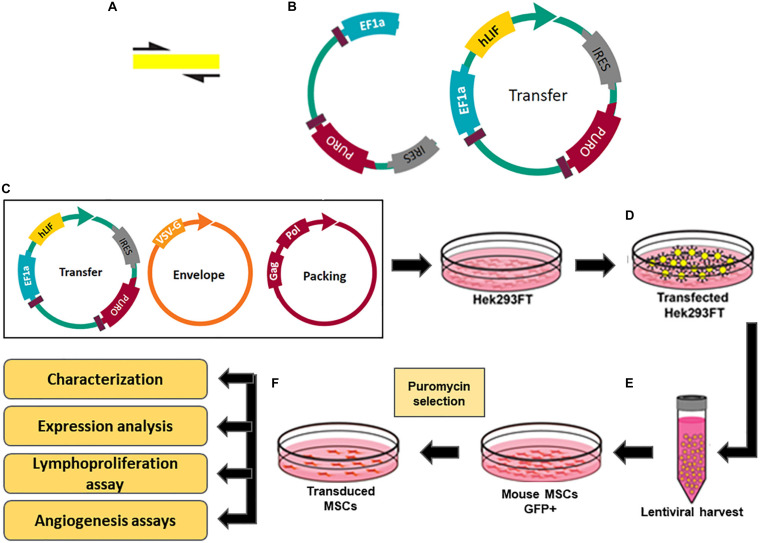
Constructs and experimental design for production of transgenic MSC overexpressing hLIF. **(A)** Amplification of coding region of hLIF; **(B)** Design of pEGIP vector and hLIF lentiviral transfer vector containing EEF1α constitutive promoter and puromycin selection gene. Schematic representation of transgenic MSC lines generation showing **(C)** transfection of Hek293FT cells with the lentiviral system vectors; **(D)** Production of lentiviral particles by the Hek293FT cells; **(E)** Harvest and concentration of lentiviral particles; **(F)** Transduction of MSC with lentivirus and selection of the clones overexpressing human LIF.

MSCs were transduced using non-replicative lentiviral particles carrying the gene of interest (hLIF). The second generation lentiviral system was composed of three vectors: (1) psPAX2, a plasmid packaging (Addgene, Watertown, MA, United States; plasmid # 12260); (2) pMD2.G, envelope protein expressing plasmid (Addgene, plasmid # 12259); and (3) pEGIP, expression vector for stable integration of GFP expression cassette with puromycin selection (Addgene, plasmid # 26777), according to the protocol previously described (Zou et al., 2009). For the generation of the lentiviral *LIF* expressing vector, the coding sequence for the gene was amplified by PCR using pUNO1-hLIFa (Invivogen, San Diego, CA, United States) as template with the primer sequences: hLIF_*Bam*HI_F-CCAACGTGACGGACTTCCC and hLIF_*Bsr*GI_R-TACACGACTATGCGGTACAGC; After cloning, the coding sequence of interest, together with the pEGIP expression vector, were submitted to confirmatory digestion with restriction endonucleases *Bam*HI and *Bsr*GI. After that, the reaction products were subjected to an electrophoretic run in 1% agarose gel in TAE 1X buffer, and the bands with the highest molecular weight, cut and purified. The amplicon was subcloned into the pEGIP vector in the *Bam*HI/*Bsr*GI GFP flanked region (Figures 1A,B). The construct was sequenced using the primer F: GGCCAGCTTGGCACTTGATGTA and R: CTAGGAATGCTCGTCAAG. The lentivirus carrying *LIF* was produced in Human embryonic kidney 293Ft (HEK 293FT) cells by transient co-transfection with PSPAX2, PMD2.G and transfer vector pEGIP containing *gfp* or *LIF* as gene inserts, in a proportion of 2:1:3, respectively, using the calcium phosphate method (Figure 1C) (Tiscornia et al., 2006). Supernatants from HEK 293FT cells were collected 48 h after transfection and centrifuged at 300 × *g* for 10 min to remove cell debris and filtered through a 0.45 μm pore-size filter. The lentiviral particles were concentrated by ultracentrifugation for 90 min at 50,000 × *g* and 4°C (Figures 1D,E). Viral titers were estimated by comparing HEK cells infection with a GFP^+^ control lentivirus, generated by the same method, in the dilutions (0,10^–1^, 10^–2^, and 10^–3^) followed by the evaluation of the percentage of GFP fluorescent HEK cells by flow cytometry after 72 h. To estimate the titers of lentiviral stocks carrying the hLIF gene and empty pEGIP (control), we applied to the formula:

$$t\,i\,t\,l\,e=\left(\frac{F\times C{}^{\circ }}{V}\right)\times D\,F$$

where *F* is the frequency of positive GFP cells after transduction; *C*^o^ is the number of cells at the time of infection; *V* is the volume of the lentiviral solution used for transduction; and *DF* is the dilution factor (White et al., 1999). The estimated titer for lentivirus stocks carrying the hLIF gene and pEGIP control was 10^7^ TU/mL. The MSCs at passage 5 were seeded in a 6-well plate and maintained in incubator until they reached 80% confluency. The transduction of MSCs was performed by incubating the cells with the lentiviral stocks at 1 MOI of pEGIP LIF or control and 6 μg/ml of polybrene for 24 h. The culture medium was changed and the cells were cultured for 48 h before adding 2 μg/ml of puromycin (Thermo Fisher Scientific) for selection (Figure 1F). The cell lines obtained, MCS_LIF (MSC transduced with LIF vector) and MSC_pEGIP (MSC transduced with control vector), as well as the wilt-type MSC, were then expanded for characterization and further experiments.

### Quantitative Real-Time PCR

Total RNA was extracted from the MSC and MSC_LIF cell lines using the TRIZOL^®^ extraction reagent (Thermo Fisher Scientific). The quantification of RNA was performed on a NanoDrop^TM^ 1000 spectrophotometer (Thermo Fisher Scientific). The purity of the samples obtained by the ratio A260 nm: A280 nm demonstrated a proportion between 1.8 and 2.0. Aliquots of 1 μg of high quality RNA were used for cDNA synthesis using SuperScript III reverse transcriptase (Invitrogen Waltham, MA, United States) after treatment with DNAse I. To analyze the expression of proangiogenic factors in MSC and MSC_LIF cells, we used the following primer sets purchased from Integrated DNA Technologies^TM^ (Coralville, IA, United States): ACTA2, TAGLN, COL4A1, CCL2, CXCL2, ANG, VEGF, and NG2 (Table 1), and Sybr Green (Thermo Fisher Scientific). The reactions were done in triplicate, and the average cycle thresholds (Ct) values were used to calculate the expression of each gene using the B2M gene as a normalizer. The PCR amplification was performed on an ABI7500 real-time PCR system (Thermo Fisher Scientific), under standard thermal cycle conditions.

**TABLE 1:** Oligonucleotide primer sequences.

Primers	Sequences 5′–3′	Amplicon (bp)
LIF_Hs_qPCR_F	CCAACGTGACGGACTTCCC	
LIF_Hs_qPCR_R	TACACGACTATGCGGTACAGC	82
ACTA2_Mm_qPCR_F	GTCCCAGACATCAGGGAGTAA	
ACTA2_Mm_qPCR_R	TCGGATACTTCAGCGTCAGGA	102
TAGLN_Mm_qPCR_F	CCTGGCCGTGAGAACTTCC	
TAGLN_Mm_qPCR_R	CCCAGGTTCATTAGTGTCCGC	189
COL4A1_Mm_qPCR_F	CTGGCACAAAAGGGACGAG	
COL4A1_Mm_qPCR_R	ACGTGGCCGAGAATTTCACC	238
CCL2_Mm_qPCR_F	CACAGATGGCCTTGATGTTG	
CCL2_Mm_qPCR_R	CTCTGGCTCAGCATGACTCC	179
ANG_Mm_qPCR_F	CCAGGCCCGTTGTTCTTGAT	
ANG_Mm_qPCR_R	GGAAGGGAGACTTGCTCATTC	109
VEGF_Mm_qPCR_F	GAGGTCAAGGCTTTTGAAGGC	
VEGF_Mm_qPCR_R	CTGTCCTGGTATTGAGGGTGG	160
NG2_Mm_qPCR_F	GGGCTGTGCTGTCTGTTGA	
NG2_Mm_qPCR_R	TGATTCCCTTCAGGTAAGGCA	132
CXCL2_Mm_qPCR_F	CCAACCACCAGGCTACAGG	
CXCL2_Mm_qPCR_R	GCGTCACACTCAAGCTCTG	108
B2M_Mm_qPCR_F	TTCTGGTGCTTGTCTCACTGA	
B2M_Mm_qPCR_R	CAGTATGTTCGGCTTCCCATTC	104

### Enzyme-Linked Immunosorbent Assay

Cell culture supernatants from MSC_LIF were collected after 24, 48, and 72 h of culture and stored at −20°C until use. hLIF concentrations were quantified by enzyme-linked immunosorbent assay (ELISA), using the DuoSet kit (R&D Systems, Minneapolis, MN, United States), according to the manufacturer’s instructions. After incubation with a streptoavidin-peroxidase conjugate (Sigma-Aldrich, St. Louis, MO, United States), the reaction was developed using H2O2 and 3,3,5,5-tetramethylbenzidine (Sigma-Aldrich) and the 450 nm wavelength light absorbance read in spectrophotometer (BioTek Instruments, Winooski, VT, United States). Six samples were analyzed per group.

### Flow Cytometry Analysis

Mesenchymal stem/stromal cell and MSC_LIF (1 × 10^5^ cells) were resuspended in 0.9% saline solution and incubated for 30 min in flow cytometry tubes with the antibodies Sca1-PE-Cy5.5 (Thermo Fisher Scientific), CD45-APC, CD44-PE (BD Biosciences, Franklin Lakes, NJ, United States), CD29-APC and CD11b-PE (Biolegend, San Diego, CA, United States), or isotype control antibodies (Thermo Fisher Scientific), diluted at 1:100 ratio. After the incubation period, the tubes were centrifuged at 300 × *g* for 5 min and cells were washed twice with PBS solution. Immunophenotyping analyzes were performed using an LSRFortessa flow cytometer (BD Biosciences). At least 50,000 events were collected and analyzed.

### Adipogenic, Osteogenic and Chondrogenic Differentiation

To evaluate the multipotency of the cell lines, the trilineage differentiation assay was performed. In brief, 1 × 10^4^ MSC and MSC_LIF cells were cultured in 24-well plates with complete DMEM (Thermo Fisher Scientific) medium. After reaching 50–60% confluence, the medium was replaced for the specific differentiation media (all from Thermo Fisher Scientific). For adipogenic induction, the cells were cultured in StemPro Adipogenesis Differentiation Kit (Thermo Fisher Scientific) during 14 days, followed by fixation in 4% paraformaldehyde and staining with Oil red solution to observe fat-filled vacuoles. For osteogenic differentiation, the cells were cultured in the StemPro Osteogenesis Differentiation kit (Thermo Fisher Scientific), with changes of half of the differentiation medium every two days. To observe the deposition of a calcium-rich matrix after differentiation, cultures were fixated in 4% paraformaldehyde and stained the cultures with Alizarin red 2%. For chondrogenic differentiation, cells were cultured for 21 days in standard chondrogenic differentiation medium StemPro Chondrogenesis Differentiation kit (Thermo Fisher Scientific). Proteoglycan synthesis was observed by staining with Alcian Blue solution after fixation with 4% paraformaldehyde. The assay was performed in triplicate, and the experiment repeated three times. The images were captured using an AX70 microscope with a digital camera (Olympus, Shinjuku, Tokyo, Japan).

### Proliferation Assay

Mesenchymal stem/stromal cell or MSC_LIF cells (5 × 10^4^/well) were cultured in 96-well plates with complete DMEM (Thermo Fisher Scientific) medium. After 72 h, 1 μCi of methyl-^3^H-thymidine (PerkinElmer, Waltham, MA, United States) was added per well and incubated for 18 h. Cell proliferation was measured as the percent of ^3^H-thymidine incorporation for MSC or MSC_LIF using a β-plate counter (PerkinElmer). The assay was carried out with six replicates and three independent experiments were performed.

### Lymphocyte Proliferation Assay

Splenocytes obtained from C57Bl/6 mice were plated in 96-well plates (8 × 10^5^ cells/well), in a final volume of 200 μL and stimulated with Dynabeads^®^ mouse T-activator CD3/CD28 (bead to cell ratio = 1:1; Themo Fisher Scientific), and co-cultivated in the presence of wild-type or transduced mitomycin-treated MSCs (1:1, 1:10, 1:100, 1:1000 MSCs:Splenocytes ratio) or with supernatant from MSC or MSC_LIF. After 48 h of incubation, plates were pulsed with 1 μCi of methyl-^3^H-thymidine (PerkinElmer) for 18 h. The cell proliferation was determined by evaluating the ^3^H-thymidine uptake using a β-plate counter (PerkinElmer). The inhibition of splenocytes proliferation was determined in relation to controls stimulated by anti CD3/CD28 in the absence of MSCs (0:1). The assay was carried out with six replicate and three independent experiments were performed.

### Preparation of Conditioned Medium

Mesenchymal stem/stromal cell and MSC_LIF were seeded in 6-well plates (1 × 10^6^ per well) and incubated in DMEM medium (Thermo Fisher Scientific) supplemented with 10% FBS and 1% penicillin/streptomycin (Thermo Fisher Scientific) until reaching 80% confluence. The culture medium was then removed and the cells were washed with PBS. The conditioned medium was obtained by incubating these cells with 1.5 mL per well of basal EBM-2 medium (Lonza Group, Basel, Switzerland) with 0.3% albumin (Sigma-Aldrich) for 72 h. The cell-free supernatants were obtained by centrifugation at 640 × *g* for 15 min at 4°C, aliquoted and stored at −80°C until use.

### HUVEC Isolation and Culturing

Primary culture and maintenance of human umbilical vein endothelial cells (HUVEC) were performed as described previously (Jaffe et al., 1973). The institutional review board of the Climério de Oliveira Maternity Hospital (CEP – Federal University of Bahia) approved the procedures (approval number 625.059). The HUVECs were cultured in EGM-2/BulletKit medium (Lonza Group) supplemented with 100 U/mL penicillin/streptomycin (Thermo Fisher Scientific) at 37°C in 5% CO_2_ and 95% air. Human umbilical vein endothelial cells were seeded on 0.1% gelatin (Sigma-Aldrich) in EGM-2/BulletKit and replaced every 2–3 days. Passages four to six of the HUVECs were used for experiments in this study.

### Scratch Wound Healing Assay

Human umbilical vein endothelial cells (2 × 10^5^ per well) were plated in 24-well plates and cultured in EGM2/BulletKit medium (Lonza Group) until they formed a confluent monolayer (about 24 h after incubation). Then, the scratch was performed by scraping the cell monolayer in a straight line, using a p200 pipette tip. Reference points were marked close to the scratches to evaluate the same field during image acquisition. Medium was exchanged to conditioned medium from MSC and MSC-LIF and the plates were incubated at 37°C for 9 or 18 h. EBM-2 medium without supplementation was used as control. Distance between the edges captured using an inverted phase contrast microscope (Leica DMi1) at three points for each well in three timepoints: after performing the scratch (time-point 0), after 9 h in incubation and after 18 h in incubation. The assay was performed in triplicates, and the experiment repeated three times. The open wound area was quantitatively measured using ImagePro Plus 7.0 software (Media Cybernetics, Rockville, MD, United States). Results are represented as a percentage of the migration area, established by subtracting the average distance values obtained at time-point 0 by the values obtained after incubation, according to the formula:

$$\%migrationarea=\frac{\left(A\,i-A\,f\right)}{A\,i}$$

Where, *Ai* represents the initial area of the wound and *Af* represents the final area of the wound after cell migration.

### Sprouting Aortic Ring Assay

All procedures were approved by the institutional review board for animal experimentation (CEUA, UFBA-2018-131). The mouse aortic ring assay was performed as described previously (Baker et al., 2012). Thoracic aortas were dissected from male 4–8 weeks-old C57Bl/6 mice. The animals were anesthetized with ketamine and xylazine solution and euthanized by cervical dislocation. The thoracic cavities were opened and the organs carefully separated to expose aortas. Each artery was cut between the anterior end, before the heart and lung insertion, and at the posterior end before branching into the iliac arteries. After removing blood and fat-cover from vessels, the dissected aortas were cut into ∼1.0 mm rings and transferred to Petri dishes with 5 mL serum-starved in DMEM overnight. Next, the aortic rings were randomly placed onto individual wells of a 48-well plate, embedded in 250 μL of fibrin gel and, after polymerization, 1 × 10^5^ MSC or MSC_LIF cells were seeded on top of the gel. Another group was performed by incubating the aortic rings with conditioned medium from MSC or MSC_LIF every 2-3 days. EGM-2/BulletKit medium was used as a positive control (PTV_CTL) and basal EBM-2 media was used as a negative control (NGV_CTL). For 7 days, daily, microvessel sprouting was observed and imaged using an inverted phase-contrast microscope (Leica DMi1). The assay was performed with five replicates per group, and the experiment repeated three times. Quantification of microvessel sprouts number was analyzed using ImagePro Plus 7.0 software (Media Cybernetics).

### *In vivo* Matrigel Plug Assay

*In vivo* angiogenesis experiments were performed as previously described by Malinda (2009). A mixture of basement membrane matrix (ice-cold phenol red-free, reduced growth factor, Thermo Fisher Scientific) and conditioned medium (0.5 mL, 9:1 proportion) was injected subcutaneously into two-months-old C57Bl/6 wild-type mice (*n* = 4). Each mouse received two implants, totaling eight plugs per group. A buffered saline was included as a negative control during the assay. After 7 days, the mice were euthanized and the plugs were excised, photographed, and processed. Quantification of blood vessels was achieved using immunofluorescent visualization of blood vessels on frozen Matrigel sections and by measuring the amount of hemoglobin (Hb) contained in the plugs. Frozen Matrigel sections were stained with rat anti-mouse CD31 antibody (R&D system, 0.125 μg/mL in BSA/normal serum solution) followed by incubation with Alexa 488-conjugated anti-rat antibody (Molecular Probes, 10 μg/mL in PBS) as described previously by Ribeiro et al. (2019). For quantitation of functional vessels formed, the plugs were homogenized in distilled water and centrifuged at 2,400 × *g* for 5 min. The supernatant was mixed with Drabkin’s reagent (Sigma-Aldrich) for measurement of Hb. After 15 min at room temperature, the absorbance of the mixture was measured at 540 nm.

### Statistical Analyses

The results of the experiments were analyzed and continuous variables are presented as mean ± SEM. The data were analyzed using Student’s *t*-test for comparisons between two groups and 2-way ANOVA, followed by Bonferroni test or ANOVA followed by Newman–Keuls test for multiple-comparison tests, using the software GraphPad Prism version 5.0 (Software Inc., San Diego, CA, United States). *p*-Values < 0.05 were considered statistically significant.

## Results

### Generation of Transgenic MSC Lines Overexpressing hLIF

**FIGURE 2:**
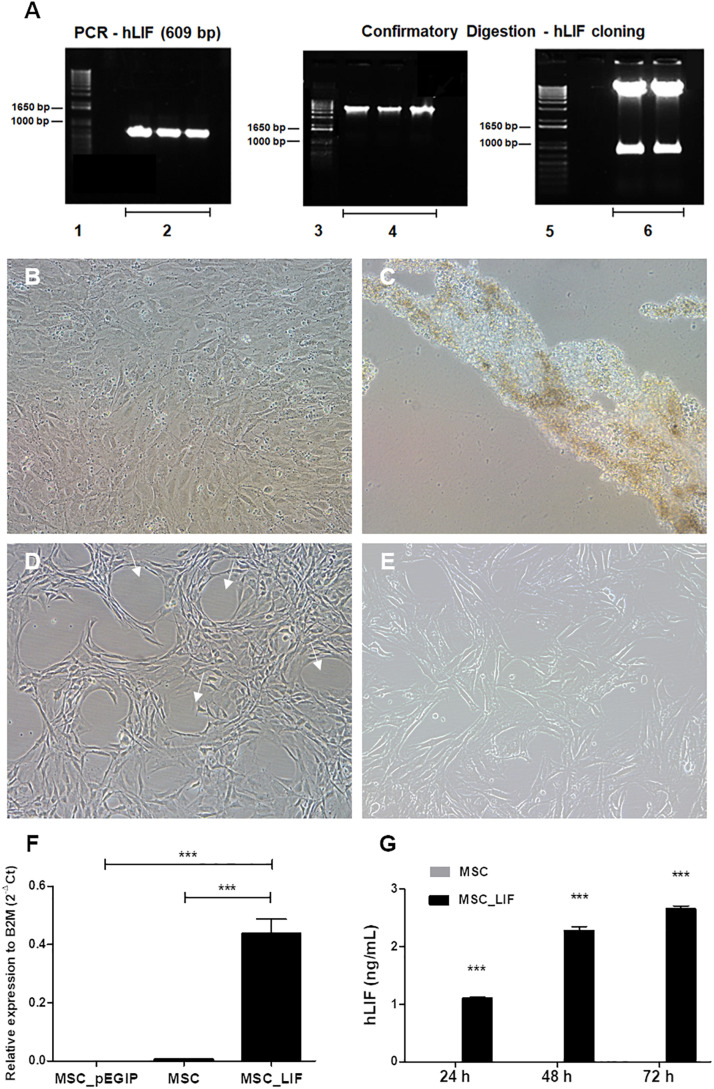
Amplification of the hLIF transgene and confirmatory digestion, antibiotic selection and establishment of stable cell cultures. **(A)** Amplification of the hLIF gene (609 bp) and confirmatory digestion of pEGIP vector after cloning (Caplan and Dennis, 2006; Murphy et al., 2013; Kim et al., 2019), molecular weight marker (Parekkadan et al., 2007), PCR reaction triplicate (Gearing et al., 1987), undigested clones (Auernhammer and Melmed, 2000), and digested clones. **(B,C)** Phase contrast images of the cell culture, showing morphology of MSC_LIF transduced culture and MSC control culture after selection with 2.0 μg/ml of puromycin, respectively. Morphology of expanded cell lines of MSC_LIF **(D)** and MSC wild-type **(E)**. The arrows highlight the halos formed in the MSC_LIF culture after transduction. 100× magnification. mRNA expression analysis of hLIF in MSC_LIF, MSC and MSC_pEGIP **(F)** and detection of the protein in the supernatant after 72 h of culture, by ELISA **(G)**. The two-way ANOVA test and the Bonferroni post-test were used to analyze the differences among the groups. Values are expressed as mean ± SEM of at least three independent experiments. ***p < 0.001.

Mouse bone marrow-derived MSC were transduced with vectors carrying *LIF* or *gfp* control vector (pEGIP). Digestion with restriction endonucleases confirmed that amplicon was properly subcloned into the pEGIP vector (Figure 2A). Transduction was well tolerated by cells, which survived and proliferated during the puromycin selection step (Figure 2B), while in non-transduced wells exposure to the antibiotic led to complete cell culture death (Figure 2C). Despite maintaining a fibroblast-like morphology, MSC_LIF cells displayed a change in their distribution in culture, showing a tendency to spontaneously organize in circular structures (Figure 2D), compared to non-transduced control cultures (Figure 2E). The analysis of human LIF expression demonstrated a high expression of the transgene in MSC_LIF cells, while the control MSC (pEGIP) and wild-type cells did not express the human LIF gene (*p* < 0.001) (Figure 2F). Furthermore, the production of the protein was confirmed by ELISA, in the supernatants of MSC_LIF cells collected after 24 h (*p* < 0.001), 48 h (*p* < 0.001), and 72 h of culture (*p* < 0.001) (Figure 2G).

### Characterization of MSC_LIF Cells

**FIGURE 3:**
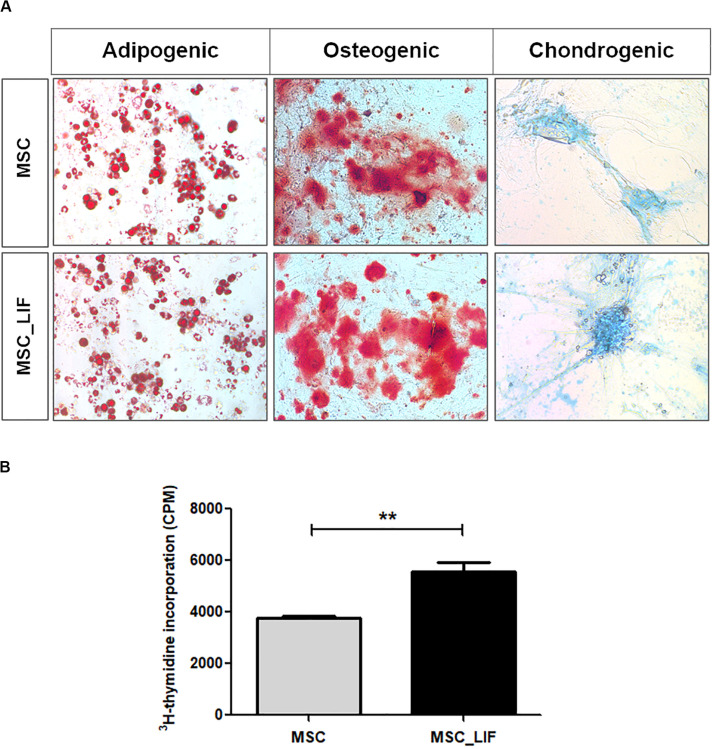
Trilineage differentiation and proliferation assays. **(A)** Cell differentiation of MSC_LIF and MSC was confirmed by staining for adipocytes (Oil red), osteocytes (Alizarin red), and chondrocytes (Alcian blue). Adipogenic and osteogenic differentiation: 200× magnification. Chondrogenic differentiation: 100× magnification. **(B)** The proliferative rate of MSC and MSC_LIF was determined by quantifying the uptake of ^3^H-thymidine after 18 h of incubation. The Student’s *t* test was used to analyze the differences between groups. Values are expressed as mean ± SEM of at least three independent experiments. **p < 0.01.

**FIGURE 4:**
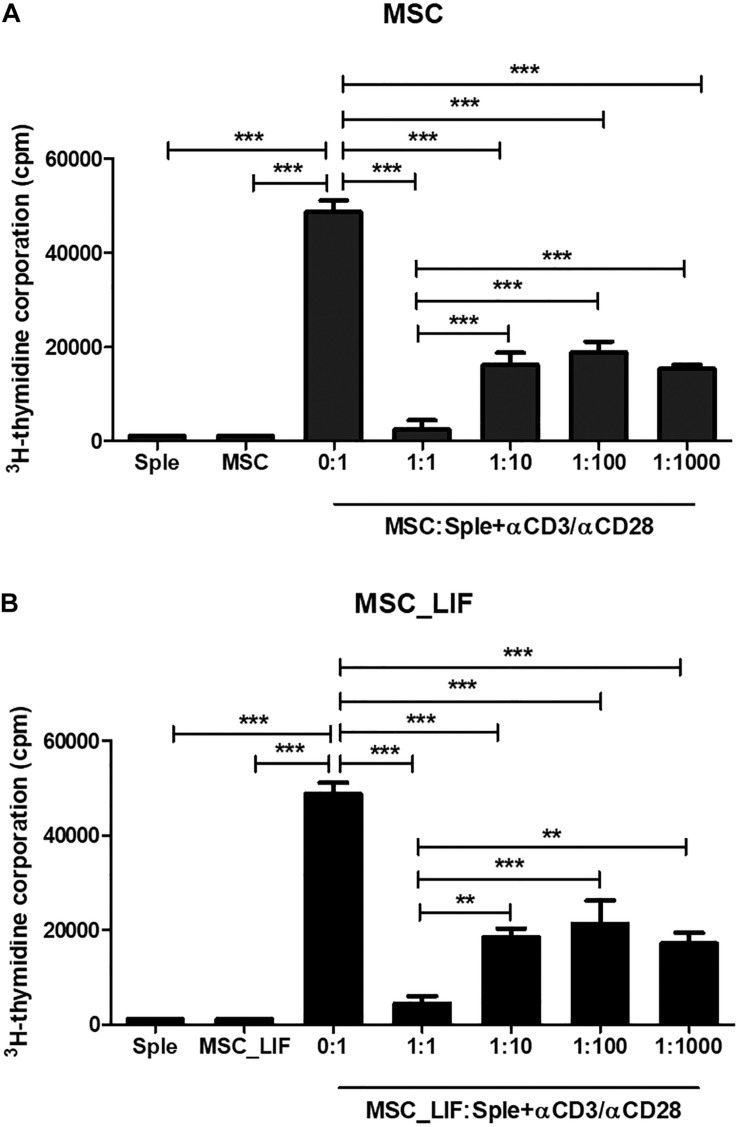
*In vitro* immunomodulatory activity of MSC_LIF. The immunomodulatory potential of MSC **(A)** and MSC_LIF **(B)** cells was determined by quantifying the uptake of ^3^H-thymidine after co-culturing these cells with splenocytes (Sple) activated with αCD3/αCD28 for 3 days. ANOVA followed by Newman–Keuls post-test were used to analyze the differences among the groups. Values represent means ± SEM of three determinations. **p < 0.01; ***p < 0.001.

The immunophenotyping of the MSC and MSC_LIF cell lines allowed to compare the expression levels of cell markers for mesenchymal cells. Similar to wild-type MSCs, transgenic MSCs showed high expression of the mesenchymal stem cell markers Sca-1, CD29 and CD44, while exhibiting low expression of hematopoietic cell markers CD45 and CD11b (Table 2). Moreover, the trilineage assay showed the maintenance of the multipotency of transduced cells, since MSC_LIF differentiated in all three lines (adipogenic, osteogenic and chondrogenic) in a similar fashion compared to the wild-type MSCs (Figure 3A). When the proliferative rat was evaluated, we found that the MSC_LIF line had an increased proliferation, when compared to the parental MSC line (*p* = 0.0029) (Figure 3B). The immunosuppressive potential of the transduced cells was also investigated by culturing splenocytes stimulated by mitogen (αCD3/αCD28), with MSC (Figure 4A) or MSC_LIF (Figure 4B) mitomycin-treated cells. The transduced MSC_LIF line, however, showed a similar immunosuppressive capacity than wild-type MSC.

**TABLE 2:** Flow cytometry analysis of cell surface markers in MSC lines.

Cell marker	MSC_wild-type	MSC_LIF
CD29	98.2%1.52	99.9% ± 0.01
CD44	97.3%2.37	96.3% ± 3.39
CD73	98.5%1.40	99.7% ± 0.05
Sca-1	95.0%5.98	99.0% ± 0.07
CD11b	0.82%0.89	0.90% ± 0.31
CD45	0.35%0.062	0.52% ± 2.04
CD34	0.1%0.99	0.85% ± 0.30

*The values represent the mean percentage ± standard deviation of two experiments.*

### Overexpression of LIF Promotes Upregulation of Pro-angiogenic Factors

**FIGURE 5:**
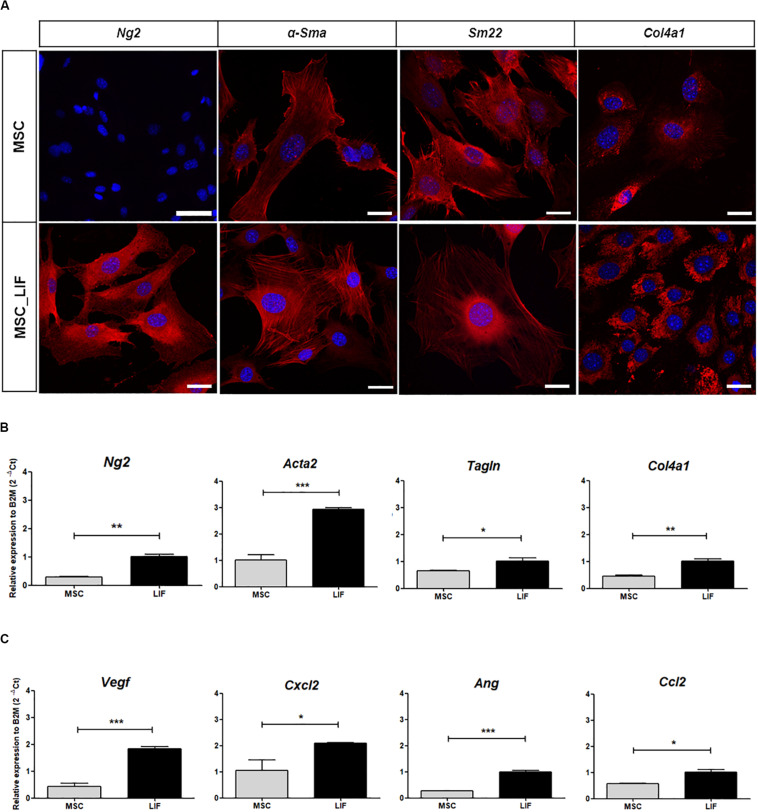
Pro-angiogenic factors expression analysis. **(A)** Immunofluorescence analysis of MSC and MSC_LIF cells with antibodies against Ng2, α-Sma, Sm22 and Col4a1, markers related to perivascular cells (all marked in red). Nuclei are contrasted with DAPI (blue). **(B)** Gene expression analysis of MSC and MSC_LIF to *Ng2*, *Acta2* (α-Sma gene), *Tagln* (Sm22 gene) and *Col4a1*. **(C)** mRNA expression analysis of the pro-angiogenic factors *Vegf*, *Cxcl2*, *Ang* and *Ccl2* (MCP-1 gene) in MSC and MSC_LIF cells. The Student’s *t* test was used to analyze the differences between groups. Values are expressed as mean ± SEM of at least three independent experiments. *p < 0.05; **p < 0.01; ***p < 0.001. Scale bars of MSC-Ng2 = 75 μm. Scale bar of other images = 25 μm.

Immunostaining and RT-qPCR array were performed to assess whether the overexpression of LIF in MSCs caused changes in the transcription of genes related to angiogenesis. Most of the proangiogenic factors and perivascular cell markers were positively regulated when compared to wild-type MSC cells. Immunofluorescence staining of MSC and MSC_LIF cells with antibodies against the perivascular cell markers showed an increased expression in MSC_LIF, when compared with MSC, for Ng2 (*p* = 0.0017), αSMA (*p* = 0.0001), Sm22 (*p* = 0.0375), and Col4a1 (*p* = 0.0032) (Figure 5A). The RT-qPCR analysis confirmed levels significantly higher in the transcripts for these markers in MSC_LIF than in control MSC (Figure 5B). In addition, MSC_LIF also showed upregulated gene expression of proangiogenic factors *Vegf* (P = 0.0001), *Cxcl2* (the functional homolog of IL-8 in mice) (*p* = 0.0114), angioginine (*Ang*) (*p* = 0.0063) and *Ccl2*, or MCP-1 (*p* = 0.0125) (Figure 5C).

### Conditioned Medium From MSC-LIF Promotes *in vitro* Endothelial Cell Migration

**FIGURE 6:**
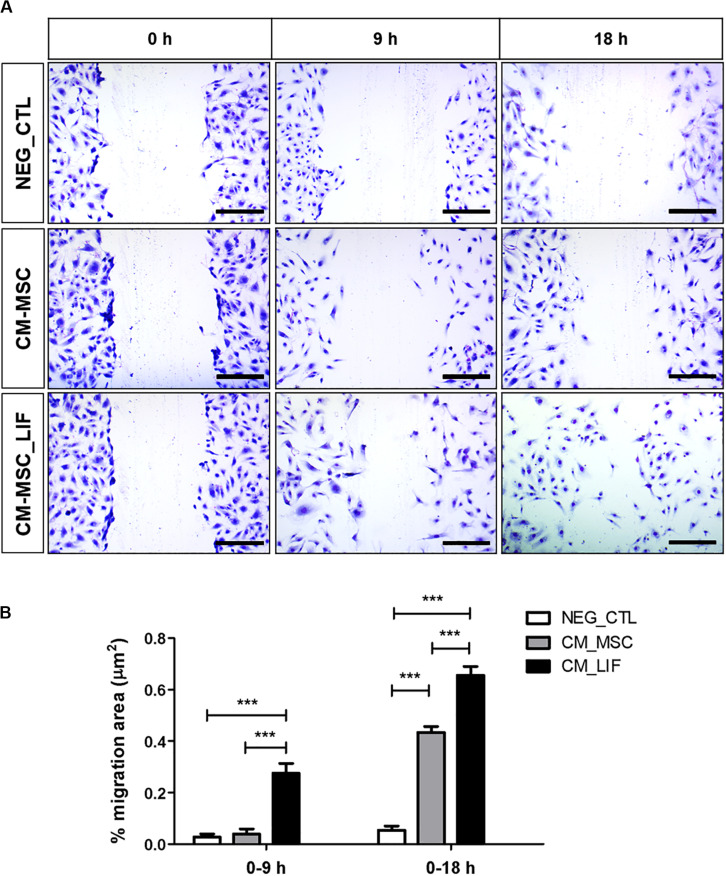
*In vitro* endothelial cell migration. **(A)** Representative image of the migration of HUVEC cells, stained with violet crystal, after 9 or 18 h of incubation in conditioned medium of MSC (CM-MSC) or MSC_LIF (CM-MSC_LIF). **(B)** Quantification of the percentage of variation in the wound area (% migration area). The two-way ANOVA test and the Bonferroni post-test were used to analyze the differences among the groups. Values are expressed as mean ± SEM of at least three independent experiments. ***p < 0.001. Scale bars = 250 μm.

**FIGURE 7:**
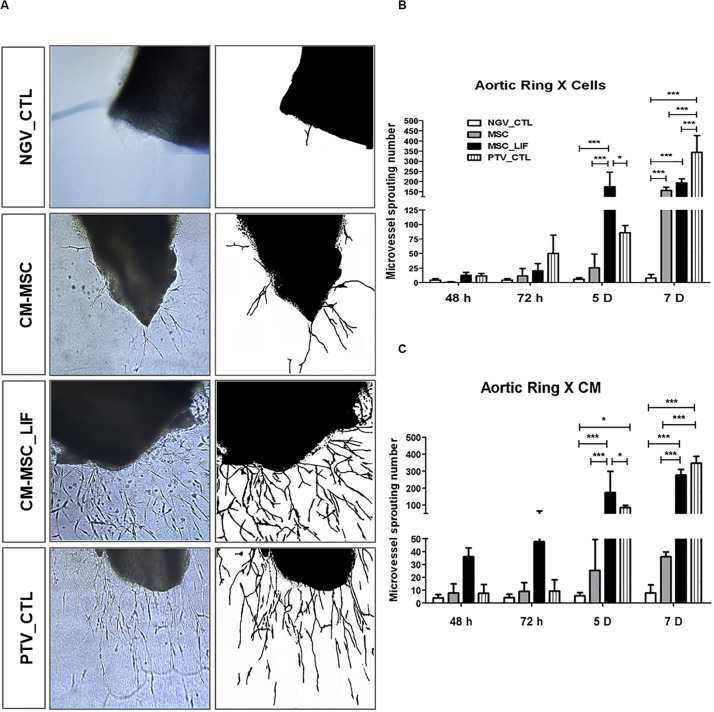
Sprouting of microvessels *in vitro*. **(A)** Representative image of microvessel growth from aortic rings after 5 days of incubation in conditioned medium of MSC (CM-MSC) or MSC_LIF (CM-MSC_LIF). The EBM-2 medium supplemented with recombinant angiogenic factors was used as a positive control (PTV_CTL), and baseline EBM-2 was the negative control (NGV_CTL). Quantification of the number of aortic ring sprouts incubated with **(B)** MSC or MSC_LIF cells or **(C)** conditioned medium from MSC or MSC_LIF on top of the fibrin gel. The two-way ANOVA test and the Bonferroni post-test were used to analyze the differences among the groups. Values are expressed as mean ± SEM of at least three independent experiments. *p < 0.05; **p < 0.01; ***p < 0.001.

Next we assessed the proangiogenic activity of MSC_LIF. As a first step toward investigating whether MSC_LIF produces paracrine factors that promote angiogenesis, conditioned medium (CM) was obtained from MSC and MSC_LIF and tested in an endothelial cell migration assay using HUVEC cells. Conditioned medium-MSC and CM-MSC_LIF conditions significantly facilitated monolayer wound closure in comparison to the vehicle control medium (*p* < 0.01). The migration of HUVECs, however, was significantly greater in the presence of CM-MSC_LIF compared to CM from control cells (Figure 6). To investigate the angiogenic effects of MSC_LIF on vascular networks, a mouse aortic ring assay was performed. Figure 7A shows the microvessels outgrowth from mouse aortic rings after 5 days of incubation. Mouse aortic explants incubated with PBS alone for 5 days, which showed no microvessel outgrowth, while the aortic rings treated with both CM-MSC and CM-MSC_LIF exhibited an increased outgrowth (Figure 7A). The total number and length of the outgrowths, however, were significantly higher in rings incubated in the presence of MSC_LIF cells compared with MSC (*p* < 0.001, at 5 days of incubation), and CM-MSC_LIF also demonstrated better performance when compared with CM-MSC (*p* < 0.0001, at 5 days of incubation) (Figure 7B). The CM-MSC_LIF stimulated the formation of tubular networks as early as 4 days following seeding onto the matrix, and the structures were maintained for a minimum of 8 days.

### MSC_LIF Promotes *in vivo* Angiogenesis

**FIGURE 8:**
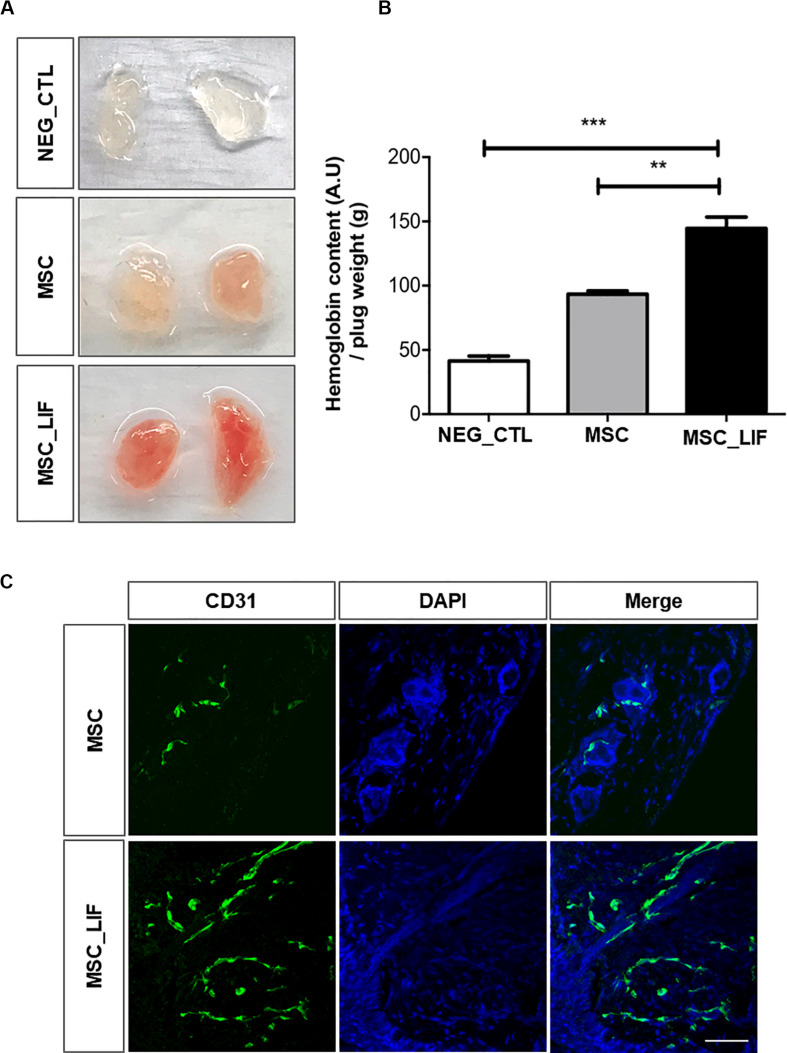
Pro-angiogenic potential of MSC_LIF conditioned medium *in vivo*. Mixture of Matrigel with indicated conditioned medium (MSC or MSC_LIF) or phosphate buffered solution (NEG_CTL) was injected into a mouse ventral area (*n* = 3). After 7 days, Matrigel plugs were measured for hemoglobin (Hb) using Drabkin’s solution. **(A)** Representative images and **(B)** quantification of Hb. **(C)** Immunofluorescent staining of frozen Matrigel section with antibodies against endothelial marker CD31 (green). Nuclei are counterstained with DAPI (blue). The two-way ANOVA test and the Bonferroni post-test were used to analyze the differences among the groups. Values are expressed as mean ± SD of at least three independent experiments. **p < 0.01; ***p < 0.001. Scale bars = 50 μm.

The effects of CM-MSC and CM-MSC_LIF on *in vivo* angiogenesis were also examined with a Matrigel plug assay. The induction of new blood vessel formation was not evident in the Matrigel plug containing PBS alone. By contrast, the inclusion of either CM significantly induced the development of new blood vessels. The Hb content normalized to the weight of the analyzed Matrigel plug was significantly higher in CM-MSC_LIF plugs in comparison to CM-MSC or PBS plugs (0.5 and 3.0 fold higher, respectively) (Figures 8A,B). The immunofluorescence staining of Matrigel sections with antibody against endothelial marker showed CD31-positive vascular structures, as well as clusters of individual endothelial cells (Figure 8C). A higher density of blood vessels was visualized on CM-MSC_LIF plugs in relation to CM-MSC plugs. Thus, CM-MSC_LIF was more potent than CM-MSC to induce ingrowth of new blood vessels into the Matrigel implant.

## Discussion

The main therapeutic properties of MSCs are attributed to their ability to secrete a series of soluble bioactive molecules, such as cytokines and growth factors, important in the regulation of various biological processes, including angiogenesis (Zhang et al., 2014). With several techniques now available that allow MSCs to be easily manipulated genetically, these cells became excellent vehicles for gene therapy (Porada et al., 2013). In the present study we described the generation of a MSC cell line genetically modified with a lentiviral vector to overexpress LIF.

Several methods have been employed to induce genetic modifications in MSCs, including those using viral vectors or non-viral vectors. Gonçalves et al. (2018) showed that the lentivirus-based transduction method used here is highly efficient and allowed to achieve high levels of expression of the interest transgene without harming the intrinsic properties of the mesenchymal cells (Gonçalves et al., 2018). In our study, the cells obtained were able to produce and secrete hLIF, in addition to maintaining the main characteristics of MSCs, such as the specific immunophenotype, differentiation potential, and immunosuppressive activity. In addition, an increase in the proliferative capacity of MSC_LIF was observed, indicating that the proliferative stimulus of LIF in some cell types can also be seen in MSC (Nicola and Babon, 2015).

Interestingly, the MSC_LIF transcriptomic analysis revealed the positive regulation of four main factors (VEGF, IL-8, MCP1, and Ang) related to vascular regeneration and increased pro-angiogenic potential. The role of LIF in the angiogenic process remains poorly understood. Studies focusing on the influence of LIF on the vascular development have demonstrated that its effects are highly pleiotropic, concentration-dependent and cell-specific for successful angiogenesis. Recently, Mohri et al. (2006) demonstrated that LIF acts at different stages of the mesoderm development process, including the speciation of hematopoietic and endothelial cells and the development of the cardiovascular system. Furthermore, stimulation of Sca-1^+^ cardiac stem cells with recombinant LIF resulted in their endothelial differentiation. Parallel to this, Paradis and Gendron (2000) showed that the combined effect of LIF and basic fibroblast growth factor (bFGF) can promote the formation of capillary structures in the EMI embryonic endothelial cell line. In both studies, activation of the STAT3 pathway by LIF was closely related to endothelial differentiation (Paradis and Gendron, 2000; Mohri et al., 2006). Altogether, these studies reinforce the pro-angiogenic potential of LIF.

Previously, Do Rhee et al. (2004) showed that signaling components JAK2, Tyk2, Erk1/2, STAT1, and STAT3 were co-expressed with LIF receptor in perivascular and stromal cells, suggesting that LIF have an effect on other cells, rather than endothelial cells, during vascular development. In our study, LIF transgenic expression did not induce endothelial cell phenotype or expression of endothelial-cell markers (CD31) on MSC, but rather induced the expression of perivascular characteristic markers (Ng2, Acta2, Col4, Tagln). This pattern of transcript expression was confirmed by immunofluorescence staining. In agreement, MSCs derived from human limbal stroma demonstrated increased expression of Pdgfr-b and Acta2, suggestive perivascular markers, and downregulation of endothelial markers (CD31, CD34) when cultivated and expanded in the presence of LIF and b-FGF (Li et al., 2012). In addition, pericyctic populations show high expression of cytokines and immunoregulatory chemokines, including LIF (Gaceb et al., 2018). In muscle pericytes, LIF is related to cardioprotective effects. The transplantation of skeletal muscle pericytes in a model of acute myocardial infarction improved cardiac function by reducing hypoxia and increasing angiogenesis due to increased expression of molecules such as IL-6 and LIF (Chen et al., 2013). Future studies are needed to investigate how LIF transgenic expression might promote perivascular characteristic markers without changing their mesenchymal identity and phenotype.

Recent studies have investigated the applicability of mesenchymal stem cells to ischemic diseases, such as peripheral arterial disease, myocardial infarction and stroke (cerebral ischemia) (Bronckaers et al., 2014). However, many studies have reported on heterogeneous proangiogenic properties of MSC as a bottleneck to improve MSC-based therapies and clinical utility (Du et al., 2016; Pinto et al., 2020). Recently, Kim et al. (2019) have identified the key factors secreted by MSC that correlate to vascular regenerative efficacy in the treatment of ischemic diseases. These trophic factors are considered efficient biomarkers for predicting the proangiogenic effects of MSCs. In our study, we demonstrated that LIF overexpression was correlated with statistically significant upregulation of four genes *Vegf*, *Ccl2* (functional IL8 homolog in mice), *Cxcl2* (Mcp1 gene) and *Ang*, indicating the proangiogenic potency of LIF_MSCs. Several studies have reported LIF signaling on gene expression during differentiation of MSCs, but the role of LIF on expression of proangiogenic growth factors and cytokines has not been demonstrated and deserves attention in future investigations (Oskowitz et al., 2008; Wang et al., 2018, 2019).

In our study, we showed that increased expression of LIF appears to have enriched the secretome of MSC with angiogenic growth factors. Thus, the conditioned medium derived from MSC_LIF (CM-MSC_LIF) stimulated *in vitro* migration of endothelial cells in a conventional two-dimensional assay and enhanced angiogenic sprouting in a three-dimensional model using large vessel (aortic) explants. A similar effect was also observed during co-culture of CTM_LIF with aortic tissue. Finally, in *in vivo* Matrigel assays, the plugs with CM-MSC_LIF displayed significantly higher level of Hb content and were positive stained for CD31-vessels in contrast to control Matrigel plugs. Altogether, these data showed that conditioned medium derived from LIF_MSCs displayed higher proangiogenic activities in comparison to conditioned medium derived from control MSCs.

There is conflicting data on the role of LIF in angiogenesis in the literature, both *in vitro* and *in vivo*. The protective effect of LIF on vasculature by regulating normal vessel density and thickness have been documented in the retina (Yang et al., 2018) while several investigators have reported contradictory effects of LIF on normal and pathological vascular development (Ash et al., 2005; Liu et al., 2019). Liu et al. (2015) reported that LIF stimulates the *in vitro* growth and expansion of Human corneal endothelial cells (HCECs) by blocking contact inhibition via activation of the LIF-Janus kinase 1 signal transducer (JAK1) and transcription signaling activator 3 (STAT3) (Liu et al., 2015). The retinas of LIF-deficient mice display increased microvessel density due to increased VEGF expression in the vascularized area (Kubota et al., 2008). However, exogenous LIF inhibits VEGF-mediated angiogenesis *in vitro* in bovine aortic endothelial and bovine microvascular endothelial cells (Pepper et al., 1995). Together, these studies have shown that LIF modulates VEGF expression through different mechanisms to ensure adequate capillary density.

In the present study, VEGF and other driving angiogenic factors showed increased transcript expression levels in LIF-overexpressing MSCs, while its conditioned medium showed enhanced proangiogenic potential. These data suggest that the secretory signatures of MSC_LIF and consequently, the therapeutic efficacy of conditioned medium from MSC_LIF can be modulated by LIF overexpression. We propose that the secretion of LIF by LIF-expressing MSCs could enhance its proangiogenic effect. Here we found that transcript levels of proangiogenic biomarkers that promote vascular regeneration were increased in response to LIF overexpression, although the molecular mechanisms involved in this process and the secreted factors were not investigated here.

Finally, our study demonstrated that the transgenic expression of LIF in mouse bone marrow-derived mesenchymal stem cells determined a higher proangiogenic activity, via positive regulation of biomarkers related to angiogenic growth. However, the mechanisms of LIF signaling capable of modifying the secretome of MSCs and inducing angiogenesis in ECs remain poorly understood, requiring further investigation. In addition, the therapeutic potential of MSC_LIF in a model that includes all the pathophysiological aspects of ischemic diseases still needs to be investigated.

## Data Availability Statement

All datasets generated for this study are included in the article.

## Ethics Statement

The studies involving human participants were reviewed and approved by CEP-Federal University of Bahia. The patients/participants provided their written informed consent to participate in this study. The animal study was reviewed and approved by CEUA-Hospital São Rafael.

## Author Contributions

GCS, DS, VF, BS, IO, TS, BP, and GLS contributed to the conception and design, collection and/or assembly of data, data analysis and interpretation, and manuscript writing. RR contributed to the conception and design and financial support. MS contributed to the conception and design, data analysis and interpretation, and manuscript writing. All authors read and approved the final version of the manuscript.

## Conflict of Interest

The authors declare that the research was conducted in the absence of any commercial or financial relationships that could be construed as a potential conflict of interest.
